# Supplementary material for: A phase transition enhances the catalytic activity of SARM1, an NAD+ glycohydrolase involved in neurodegeneration
Source: eLife. 2021 Jun 29;10:e66694. doi: 10.7554/eLife.66694 (PMC8266388; doi:10.7554/eLife.66694)
Supplement: Supplementary file 1. — (A) The Steady-State Kinetic Parameters for Pure Wild Type SARM1 TIR (10 µM) with Varied PEG. (B) The Steady-State Kinetic Parameters for Pure Wild Type SARM1 TIR with Varied Enzyme Concentration. [file elife-66694-supp1.docx]

| **Supplementary File 1a. The Steady-State Kinetic Parameters for Pure Wild Type SARM1 TIR (10 µM) with Varied PEG** | | | | | | | |
| --- | --- | --- | --- | --- | --- | --- | --- |
|  | 0% | 10% | 12.5% | 15% | 17.5% | 20% | 25% |
| *K*_m_ (µM) | 1750 ± 100 | 650 ± 100 | 640 ± 100 | 390 ± 70 | 440 ± 80 | 390 ± 60 | 500 ± 100 |
| *k*_cat_ (s^-1^) | 0.00037 ± 0.00007 | 0.20 ± 0.02 | 0.37 ± 0.03 | 0.45 ± 0.03 | 0.59 ± 0.04 | 0.59 ± 0.03 | 0.86 ± 0.06 |
| *k*_cat_/*K*_m_ (M^-1^s^-1^) | 0.21 ± 0.05 | 300 ± 80 | 590 ± 100 | 1200 ± 300 | 1300 ± 300 | 1500 ± 300 | 1700 ± 400 |
|  | | | | | |  |  |

| **Supplementary File 1b. The Steady-State Kinetic Parameters for Pure Wild Type SARM1 TIR with Varied Enzyme Concentration** | | | | | | | |
| --- | --- | --- | --- | --- | --- | --- | --- |
|  | 500 nM  PEG | 1 µM  PEG | 2.5 µM  PEG | 5 µM  PEG | 10 µM  PEG | 10 µM | 20 µM |
| *K*_m_ (µM) | 1600 ± 600 | 800 ± 30 | 230 ± 30 | 380 ± 70 | 500 ± 90 | 1700 ± 600 | 1200 ± 200 |
| *k*_cat_ (s^-1^) | 0.24 ± 0.04 | 0.19 ± 0.03 | 0.51 ± 0.01 | 0.68 ± 0.04 | 0.86 ± 0.06 | 0.0004 ± 0.0001 | 0.0023 ± 0.0002 |
| *k*_cat_/*K*_m_ (M^-1^s^-1^) | 150 ± 70 | 240 ± 10 | 2200 ± 30 | 1800 ± 70 | 1700 ± 70 | 0.24 ± 0.01 | 1.9 ± 1 |
|  | | | | | |  |  |
